# Comparison of Piezoelectric and Optical Projection Imaging for Three-Dimensional In Vivo Photoacoustic Tomography

**DOI:** 10.3390/jimaging5010015

**Published:** 2019-01-11

**Authors:** Robert Nuster, Günther Paltauf

**Affiliations:** Department of Physics, University of Graz, 8010 Graz, Austria

**Keywords:** photoacoustic imaging, optoacoustic imaging, ultrasound array, piezoelectric polymer, phase contrast, in vivo imaging, projection imaging

## Abstract

Ultrasound sensor arrays for photoacoustic tomography (PAT) are investigated that create line projections of the pressure generated in an object by pulsed light illumination. Projections over a range of viewing angles enable the reconstruction of a three-dimensional image. Two line-integrating arrays are compared in this study for the in vivo imaging of vasculature, a piezoelectric array, and a camera-based setup that captures snapshots of the acoustic field emanating from the sample. An array consisting of 64 line-shaped sensors made of piezoelectric polymer film, which was arranged on a half-cylindrical area, was used to acquire spatiotemporal data from a human finger. The optical setup used phase contrast to visualize the acoustic field generated in the leg of a mouse after a selected delay time. Time-domain back projection and frequency-domain back propagation were used for image reconstruction from the piezoelectric and optical data, respectively. The comparison yielded an about threefold higher resolution for the optical setup and an about 13-fold higher sensitivity of the piezoelectric array. Due to the high density of data in the camera images, the optical technique gave images without streak artifacts, which were visible in the piezo array images due to the discrete detector positions. Overall, both detection concepts are suited for almost real-time projection imaging and three-dimensional imaging with a data acquisition time of less than a minute without averaging, which was limited by the repetition rate of the laser.

## 1. Introduction

Photoacoustic tomography (PAT), which is also known as optoacoustic tomography, reveals the optical absorption properties of an object from acoustic signals generated by the absorption of modulated electromagnetic radiation. Due to its ability to provide high contrast images of naturally occurring chromophores such as hemoglobin in biological tissues, PAT has the potential to become an important biomedical diagnostic imaging modality. Most commonly, the used radiation is generated by short pulsed laser sources in the visible and near infrared spectral range, and the acoustic waves, which are caused by the thermoelastic effect, are detected by broadband ultrasound sensors. In three-dimensional (3D) PAT, the excitation laser pulses are absorbed in a large volume of the imaged object, and the broadband sensors are arranged in a way so as to gather the resulting acoustic waves over a large range of propagation directions on the surface of the object. This guarantees accurate image reconstruction from the acoustic signals. Another requirement is that the measured set of spatiotemporal data should have approximately the same size as the set of data points in the reconstructed 3D image. Data acquisition can be achieved by scanning a single detector across the surface of a sample, measuring at each position the ultrasound wave that is created by a single excitation pulse [1,2]. Since the speed of this procedure is limited by the pulse repetition rate, it is too slow for many applications, where motion artifacts can deteriorate the image quality. Therefore, alternatives are sought, using sensor arrays for parallel detection. Ideally, the sensor elements are arranged in a two-dimensional (2D) grid on an area surrounding the object. This can give the full information for a 3D image in a single shot. Devices with up to 512 elements have been demonstrated [3], but a further increase in the number of detectors makes these single-shot devices extremely complex. Therefore, a very common implementation of receiving arrays uses one-dimensional (1D) arrangements of detectors on a line or curve, which are moved relative to the imaged object and receive the imaging data over a sequence of several laser pulses. Such devices with ring-shaped arrays have been used in small animal imaging [4], and have been proposed for human breast imaging [5]. Linear and arc-shaped arrays have also been developed [6,7]. The received signals from the moving 1D array are combined, yielding a data set for 3D imaging within a few seconds. Sometimes, for instance if the linear or ring-shaped array is able to focus into a slab within the object, a 2D image of this slab can be obtained as an intermediate result from a single pulsed excitation.

For a special kind of 1D sensor array, which consists of parallel lines with a size larger than the imaged object, the reconstructed image corresponds to a projection along the line direction [8]. It has similarity with X-ray single exposure images. To form a 3D image, projection images in many directions have to be combined, such as in X-ray computed tomography. As a special case of a moving 1D array, tomography with line detectors has several benefits, such as the availability of real-time projection images enabling the observation of dynamic processes in a large volume, or the possibility to use free or guided optical beams as sensors [2,9]. Earlier, we have demonstrated a line detector array based on polyvinylidene fluoride (PVDF) sensors for the 3D imaging of phantoms and for 2D projection imaging in vivo [10]. Projection images have also been generated using optical fibers as line detectors [11]. In arrays consisting of individual sensors that measure time-dependent acoustic signals, each channel must be connected to an amplifier and an analog-to-digital converter. As an alternative that does not require external electronics but can achieve an even higher density of sensing elements, we are investigating a PAT device using a charge-coupled device (CCD) camera combined with an optical phase contrast setup for acoustic detection [12,13]. Since the acquired data structure (the CCD camera image) is 2D and gives rise to a 2D reconstruction, this concept is closely related to 1D arrays, although the pixels of the camera are arranged in a plane. While the 1D array gives pressure values as a function of time and position along the array, the CCD camera device replaces the time axis with another spatial axis, using snapshots of the acoustic field generated by photoacoustic excitation.

The optical detection of ultrasound, either using time-dependent signals [1,2,14,15] or snapshots of the acoustic field captured with a camera [13,16], has many attractive features, and has therefore been investigated as an alternative to piezoelectric detection for PAT [17,18]. Since most of the available work on PAT has focused on either of the two detection methods, direct comparisons of the two approaches are rare. The available assessments of piezoelectric versus optical sensors have focused on the performance of individual sensor elements [19,20]. The purpose of the present study is a comprehensive comparison taking into account also the image reconstruction and factors that affect the image quality. Therefore, an important part of this comparison is an evaluation of temporally continuous data acquisition at given points in space versus spatially continuous detection at given instants of time. As an imaging object, we chose in vivo vasculature, which is the most common target in PAT. We demonstrate the 3D in vivo imaging of vasculature in a human finger with the line-integrating piezoelectric array, and compare its imaging performance with the CCD camera-based setup, which was used to acquire 3D in vivo images of mouse vasculature. Upon comparison of the two devices, we point out the peculiarities of the piezoelectric and the optical techniques in terms of the image reconstruction, resolution limiting factors, possible imaging artifacts, and sensitivity.

## 2. Materials and Methods

### 2.1. Imaging Setups

**Figure 1:**
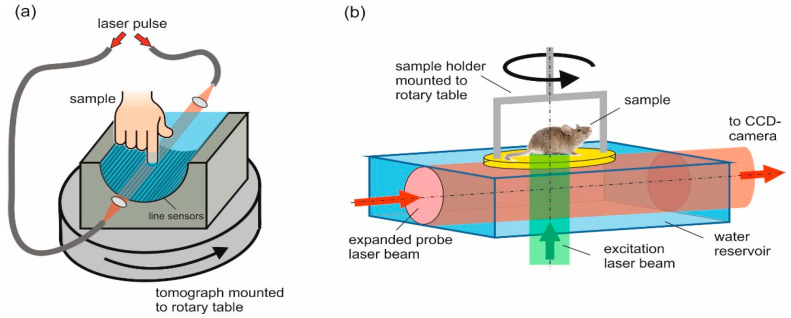
Experimental setups for photoacoustic tomography. (**a**) Piezoelectric line detector array containing 64 elements arranged on a half-cylindrical surface, rotating relative to the sample; (**b**) Optical detection using a CCD camera that takes snapshots of photoacoustic sound waves propagating from the sample into a probe laser beam. For three-dimensional (3D) imaging, the sample is rotated relative to the optical setup.

Both of the setups that are used in this work acquired 2D data sets of the photoacoustic pressure signals for the reconstruction of 2D projection images (Figure 1). To acquire 3D images, the sample had to be rotated relative to the array about an axis that was perpendicular to the projection direction. The piezoelectric line detector array was described earlier [10]. It consisted of a 110-µm thick film of the piezoelectric polymer poly (vinylidene fluoride) (PVDF) glued onto an array of 64 line-shaped copper electrodes with a width of 1.5 mm and a length of 150 mm, arranged on a half-cylindrical area with a five-cm radius. The upper side of the PVDF film was metallized and formed the common ground electrode. The bandwidth of the sensor elements was about 12 MHz [10]. The signals picked up by the line electrodes were preamplified and multiplexed into 32 channels of a digital-to-analog converter. In the previous work, 3D imaging was demonstrated on phantoms, which were mounted on a stage that rotated relative to the static array. For 3D imaging of a human finger of one of the authors, we modified the setup for the current study by mounting the tomograph on a rotary stage, allowing the acquisition of projection data of the static finger from many directions. Pulses at a wavelength of 750 nm from an optical parametric oscillator (OPO), which was pumped by the second harmonic output of a Q-switched Nd:YAG laser at a repetition rate of 20 Hz, illuminated the sample from two sides along the direction of the line sensors. The radiant exposure from each side was 13 mJ/cm². Four times averaging was used at each rotational position of the array, and the total rotation was 180° in 200 angular steps.

The homemade camera-based tomograph was also described earlier, and will only be briefly described here [13]. The mechanism of detection is the pressure-induced change of optical phase along the propagation path of a collimated, expanded probe beam through a water bath. These phase changes are made visible by a phase contrast method, causing brightness variations in the camera image that are proportional to the pressure integrated along the light path in the water bath [13]. Determined by the phase contrast optics, the magnification ratio of the imaging optics, and the pixel pitch of the CCD-camera (PCO AG, Kelheim, Germany), this method is able to detect acoustic waves in a frequency range from 1.1 MHz to 23 MHz. In vivo images of vasculature in the hind leg of a mouse were captured by fixing the mouse on a rotary stage and illuminating the leg from below with pulses from a Q-switched Nd:YAG laser at 532-nm wavelength, using a radiant exposure of 15 mJ/cm² and a repetition rate of 10 Hz. The mouse was anesthetized during the experiment to avoid motion artifacts. By using a pulsed probe laser for the phase contrast setup (527 nm, eight ns of pulse duration), the acoustic field was captured at a defined time after photoacoustic excitation. One snapshot of the acoustic field was the result of 16 images taken with the camera: eight averaged with, and eight averaged without the photoacoustic pressure distribution. The latter were used as background, and were subtracted from the former to eliminate intensity variations that were not due to the acoustic field. In this way, 100 projection images were captured, while the mouse was rotated by a total angle of 180°.

The animal experiment was approved by the Animal Protocol Review Board of the City of Vienna, and performed in accordance with the guidelines for the “Care and Use of Laboratory Animals” of the National Institute of Health.

### 2.2. Image Reconstruction

**Figure 2:**
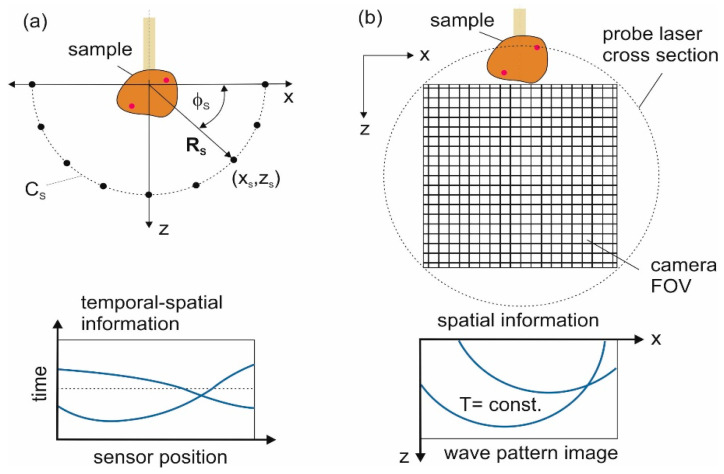
Comparison of signal acquisition methods. (**a**) Piezoelectric line detector array. The lines are arranged at positions $\left(x_{s},z_{s}\right)$ along the curve *C_s_* and are oriented in the *y*-direction. The data contain spatiotemporal information, as indicated schematically below the sensor outline; (**b**) Image acquisition with the camera-based setup. The sample is located above the field of view (FOV) of the camera, which images a part of the volume traversed by the probe laser beam. The data structure in a snapshot of the acoustic field at time *T* is purely spatial.

In both tomographic setups, the detection has the effect of integrating the pressure field along a line. The sensor elements of the piezoelectric line array are arranged on a half-cylindrical area and receive data $p(\varphi_{s},t)$, where $\varphi_{s}$ is the angular position of points (*x_s_*, *z_s_*) on the detection curve *C_s_* (an arc with radius *R_s_* spanning 180°) (Figure 2). The recorded pressure signals are given by the integral of the incoming pressure field along line direction *y* over the length *L* of the lines:

(1) $$\begin{array}{l} p(\varphi_{s},t)=\int_{0}^{L}p(\varphi_{s},y,t)\;dy\\ x_{s}=R_{s}\;\cos(\varphi_{s}),\;z_{s}=R_{s}\sin(\varphi_{s}) \end{array}$$

The camera captures snapshots of the acoustic field at a time *T*, $p_{T}(x,z)\equiv p(x,z,t=T)$, which are integrals over a length *L* along the *y*-direction of the probe beam:

(2) $$p_{T}(x,z)=\int_{0}^{L}p_{T}(x,y,z)\;dy,$$

This is also a 2D data set, but the time axis is now replaced by a second spatial axis. Therefore, a single snapshot should contain sufficient information for the 2D reconstruction.

The final result, the 3D reconstruction of the initial pressure distribution is the same for both methods; meanwhile, the detailed algorithms differ for the spatiotemporal and purely spatial data. For the piezoelectric line array, temporal pressure signals $p(\varphi_{s},t)$ are used to reconstruct the 2D distribution of initial pressure, $p_{0}(x,z)\equiv p(x,z,t=0)$. Various methods are available for this task, such as back projection [8,21], time reversal [22], or frequency-domain algorithms [8,23]. Back projection reconstructs an estimate of the initial pressure at a point by summing up the contributions from all of the detectors measured at a time that corresponds to the time of flight from the detector position to the reconstructed point:

(3) $$p_{0}(x,z)=\sum_{i=1}^{N}w_{i}\;b(x_{s,i},z_{s,i},t=d_{i}/c_{s})$$

Here, $\left(x_{s,i},z_{s,i}\right)$ denotes the position of the *i*th detector in the array, *c_s_* is the speed of sound, and $d_{i}$ is the distance from the reconstructed point (x,z) to $\left(x_{si},z_{si}\right)$. The quantity $b(x_{si},z_{si},t)$ is a function of the measured pressure signal, and weight factor $w_{i}$ contains quantities such as the angle occupied by the *i*th detector on the detection curve with respect to the reconstructed point [21,24].

Reconstruction of the 2D initial pressure distribution from the camera images uses back propagation in frequency space:

(4) $$p_{0}(x,z)=FT^{-1}[FT[p_{T}(x,z)]\cdot 2\cdot \cos(c_{s}\cdot k\cdot T)],$$

where *FT* and *FT*^−1^ are a spatial Fourier transform pair and the cosine factor has the effect of a forward and backward time propagator in which *k* is the acoustic wave number [13]. From the two obtained propagated wave fields, only the one that propagated back to *t* = 0 is used.

The final step is common for the two presented methods and involves an inverse Radon transform that is applied to projection data. In order to collect sufficient information for this reconstruction, projections $p_{0}^{\alpha }(x,z)$ have to be recorded for a set of angular positions *α*, covering at least 180°.

(5) $$p_{0}(X,Y,Z_{j})=R^{-1}\left[p_{0}^{\alpha }\left(x,z_{j}\right)\right]$$

Here, *R*^−1^ is the inverse 2D Radon transform. Upper case letters denote coordinates relative to the object, and the index *j* indicates a defined *Z*-plane to which the inverse Radon transform is applied.

## 3. Results

**Figure 3:**
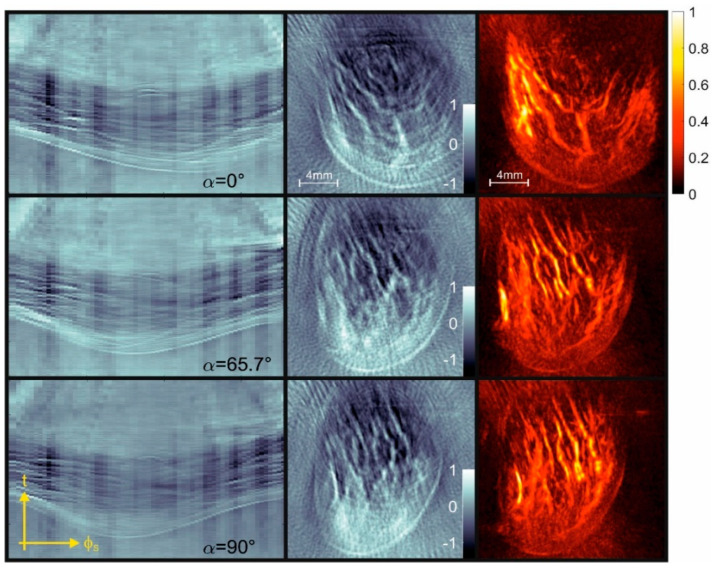
Three-dimensional photoacoustic tomography (PAT) of a human finger at a 750-nm wavelength acquired with the piezoelectric line detector array. Left column: raw data representing recorded pressure as a function of time and angular detector position on the half-cylindrical array for different orientations *α* of the tomograph relative to the finger. Second column: Projection images of the initial pressure distribution, reconstructed from the raw data displayed in the first column. Third column: Maximum amplitude projections of the 3D reconstructed image.

Both imaging setups derive 3D reconstructions from photoacoustic projection images taken along a series of observation directions. For each method, we show in the following the raw data, projection images, and maximum amplitude projections (MAPs) for selected angles. The projections are directly reconstructed from the respective raw data, whereas the MAPs are the result of the full 3D reconstruction, which requires all of the angular views as input. Figure 3 shows the results that were obtained with the piezoelectric line detector array from a human finger. The left column displays the $p(\varphi_{s},t)$ data, the center column displays the projection images, and the right column displays the MAPs.

**Figure 4:**
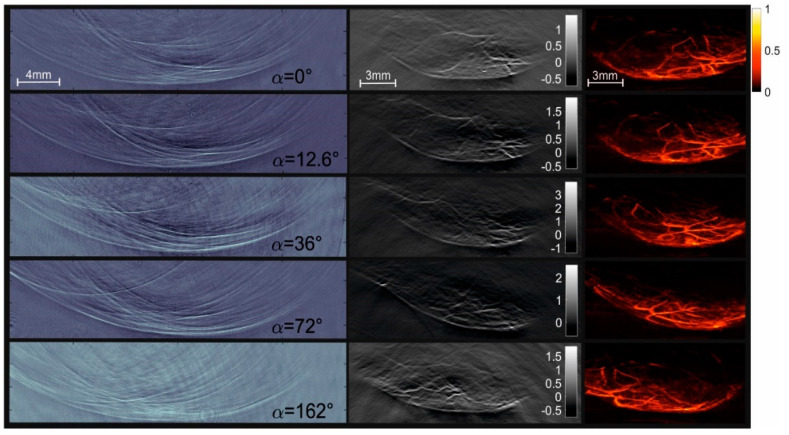
Three-dimensional PAT of vasculature in the hind leg of a mouse acquired with the camera setup. Left column: Images of the acoustic field captured at a delay time of 10 µs at various orientations *α* of the mouse relative to the optical probe beam. The second column shows the reconstructed projection images, and the third column shows the maximum amplitude projections of the three-dimensional reconstruction. Results displayed in a row are for the same orientation of the object.

In vivo images of the mouse vasculature are shown in Figure 4, displaying again raw $p_{T}^{\alpha }(x,z)$ images, reconstructed projections, and MAPs in three columns. Comparing the raw signals of the two methods clearly shows the different data structure. The camera images display circular arcs for small acoustic sources, whereas the structures in the piezo array signals have roughly a sinusoidal shape. Projection images and MAPs have similarities, since they are projections of the same object in the same viewing direction. However, there are also some differences. For instance, in the projection images, the orientation of objects is important, mainly of elongated objects such as the blood vessels. An example can be seen in the mouse images, where the projection at 72° shows a bright spot near the upper left corner. This structure is generated by a blood vessel that happened to be oriented along the direction of the projection (the *y*-axis). Since this is just one of many similar vessels in the 3D reconstruction, it does not light up any more in the corresponding MAP image. In the finger images taken with the piezoelectric array, the blood vessels are mainly oriented along the z-direction, and therefore, no such orientation-dependent enhancement occurs in the projections. Due to the relatively low number of piezo detectors, the projections contain streak artifacts. The projections reconstructed from the camera images, on the other hand, are based on spatially continuous data, and do not show such artifacts.

## 4. Discussion

The purpose of this study was to compare two different approaches—temporally continuous piezoelectric and spatially continuous optical detection—for obtaining 3D photoacoustic tomography images of vasculature in vivo. The term “continuous” is used here to indicate that in the first case, the time-dependent pressure signals are highly densely sampled, and in the second case, the spatial maps of the acoustic field are highly densely sampled. This leads to different methods in the first stage of image reconstruction, where the projections of the initial pressure distribution are obtained from the raw data. In the following, we discuss several aspects that are important in the design of a PAT device, pointing out the strengths and weaknesses of the two approaches.

### 4.1. Image Resolution

The image resolution for projections reconstructed from camera images is mainly limited by the size of the point spread function (PSF) in the raw images, and ultimately by the pixel size appearing in the object plane or the propagation distance within the camera exposure time of eight nanoseconds, whichever is larger. For our setup, we found a value of about 80 µm in the *x*-direction and *z*-direction [13]. For the cylindrical piezoelectric array, the line width limits the resolution in the angular direction, with the angular blur being minimal for points near the center of the array and increasing for points nearer to the detector [25,26,27]. The resolution in radial direction is influenced by the temporal resolution of the sensor and thus its bandwidth. Analysis of the resolution revealed values of 220 +/− 50 µm in all directions within an imaging volume that is centered at the cylinder axis and has a diameter of about 25 mm [10].

### 4.2. Sensitivity

Piezoelectric sensors are known to have a sensitivity that scales with their area. This is true as long as the incoming pressure wave hits the entire detection area at the same time, e.g., for a plane wave at normal incidence, where acoustic displacements always constructively interfere. The strength of optical sensors, on the other hand, is a sensitivity that is independent of the size of the detector spot [19]. This relation between sensor size and sensitivity has to be reconsidered for integrating acoustic sensors. An integrating line sensor, which by definition has a length that exceeds the size of the acoustic source, does not fulfill the requirement of solely constructive interference. Assuming a photoacoustic point source, the characteristic response of a line sensor is the result of the acoustic spherical wave propagating through the detector line. During this intersection, the positive and negative pressure components of the wave add up constructively and destructively in the sensor volume. The signal, which is the generation of net charges for the piezoelectric sensor or a net optical phase shift for the optical sensor, is the result of constructive interference on parts of the line. For the spherical wave, this mainly happens at the instant where the wave front just touches the line. Since the photoacoustic sound wave is always bipolar, the received signal amplitude decreases asymptotically toward zero when the wave front has passed through the detection line. The sensitivity can be characterized by the amplitude of the wave hitting the sensor line, which causes a noise equivalent signal. For instance, for the piezoelectric line sensor, a value of 20 Pa was found for normal incidence [10]. However, for the comparison of different kinds of line sensors, it is more relevant to characterize the integrated pressure signal that can be distinguished from the noise background, giving rise to a noise equivalent pressure-length product (NEPLP). For the PVDF line sensor used in the tomograph, this gives a NEPLP of 400 Pa·mm. This value considers that the object for measuring the sensitivity, which was a cylindrical volume of absorbing dye solution irradiated by a 20-mm diameter laser beam, created a pressure amplitude of 20 Pa constructively interfering over a length of 20 mm on the sensor line. Although this simple multiplicative relation seems obvious, it was double-checked by simulating the response of line detectors to the photoacoustic signal generated by a Monte Carlo-simulated energy density distribution in the phantom containing the cylindrical absorber. The sensitivity was derived from the asymptotic value of the pressure-length product when the length of the sensor increased. In a similar way, the sensitivity of the camera setup was characterized, giving a value of 5.1 kPa·mm [13]. In contrast to the piezoelectric sensor, the sensitivity of the optical method does not depend on the direction of the incoming wave. The piezoelectric tomograph has a clear sensitivity advantage. However, for image generation also, the reconstruction has to be considered. In the time-domain back projection that was applied to the piezoelectric signals, a point in the reconstructed *p*_0_ distribution is the result of a coherent sum of temporal signals from the 64 sensors in the array. The projection image reconstructed from the camera images uses the sum of all of the recorded values from all of the pixels that have been hit by the wave coming from a specific point. The number of pixels, which can be seen as separate array elements, contributing to a reconstructed point in this way depends on the time *T* at which the instantaneous acoustic field is captured, and can easily reach values on the order of 1000. This could lead to an up to eight times versus 30 times improvement of the signal-to-noise ratio after reconstruction, mitigating the sensitivity difference between the two approaches.

### 4.3. Artifacts

In addition to being spread out over many pixels, the information contained in the camera images is almost continuous or at least very densely distributed over the FOV. By contrast, the information collected by the PVDF sensor array is limited to the 64 array elements with gaps between them. This leads to streak artifacts in the images generated by the piezo array, which are not visible in the projections reconstructed from the camera data.

**Figure 5:**
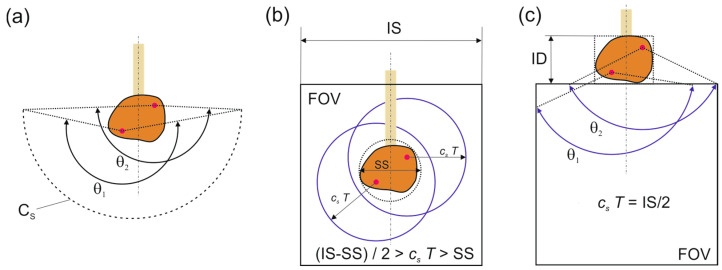
Detection angle *θ* of the different setups, which is defined as the angle covered by the detector array with respect to a point in the object. (**a**) The half-cylindrical piezo array covers a detection angle of about 180° for an object centered at its axis; (**b**) For an object submerged in the camera FOV, the detection angle can reach 360°, except for a small range that is covered by the sample holder, if the delay time *T* is chosen correctly relative to the sample size (SS) and the image size (IS); (**c**) If the sample is located above the FOV, the detection angle can reach 180° for points close to the upper boundary. The selection of *c_s_ T* = IS/2 gives similar detection angles for all of the points within the imaging depth (ID).

A comparison of projection images in Figure 4 and Figure 5 shows pronounced negative values in the images generated by the piezoelectric array. The images obtained from the camera data also show some negative values, but with lower amplitude. As negative values are unphysical and should not occur in an exact reconstruction, they must be due to some deficiencies in the data acquisition. The PVDF sensor with its layered structure has a transfer function with minima at 8 MHz and 20 MHz [10], and therefore gives an output signal that does not exactly follow the acoustic pressure arriving at the detector. The optical setup, on the other hand, is able to produce data that resemble the actual pressure distribution more closely.

A possible source of artifacts is the limited detection angle, which is defined as the range of directions over which acoustic signals emanating from a point in the object are captured by the detector array. For a 2D reconstruction with good visibility of all of the object boundaries, the detection angle should be at least 180°. Limited view effects occur if the detection angle significantly drops below 180°, leading to a deterioration of lateral resolution [28]. For the camera setup, the detection angle depends on the placement of the object relative to the field of view and the time *T* at which the image of the acoustic field is captured. If the sample is located within the FOV, waves traveling from the sample in almost all of the directions are detected [12]. This situation is shown in Figure 5b. It is limited to relatively small objects, which occupy only a fraction of the FOV. In the current setup, the photoacoustic signals were generated near the surface of an object that was larger than the FOV; thus, the requirement was to capture waves propagating into the half-space adjacent to the irradiated surface. Therefore, the imaged object was mounted above the FOV [13]. The optimum delay time is in this case the one at which the acoustic waves propagating in the largest possible detection angle have arrived in the FOV. As is shown in Figure 5c, the detection angle can achieve a value of almost 180° for a point in the object located just above the FOV. The piezoelectric half-cylindrical array, on the other hand, achieves a detection angle close to 180° for a sample located near the center of the arc, as indicated in Figure 5a. For points lying within the half-cylindrical detector area, it even exceeds 180°. The excess angular range causes some redundancy, which again causes artifacts that are largely eliminated by weight factors contained in $w_{i}$ in Equation (3) [24].

### 4.4. Operation

Probably one of the most important properties of an imaging device that is designed for preclinical research or clinical diagnostics is its ease of operation. At first sight, the piezoelectric tomograph has an advantage, since it does not require any adjustments before it is ready to use. The camera setup, on the other hand, is prone to misalignments of its optics, which can strongly deteriorate its performance. However, once it is optimized, the alignment of the phase contrast setup is quite robust against environmental perturbations, and does not need the active stabilization that other interferometric devices do. Another requirement for good performance is the optical quality of the water bath. Floating particles, but also small temperature gradients, show up in the recorded phase contrast images, and can only be eliminated to a certain degree by background subtraction. The imaging time is given by the total number of laser pulses divided by the pulse repetition rate. Using the camera setup, a single raw image was formed by taking a difference image with and without acoustic generation. With eight times averaging for each image and 100 angular orientations, the total number of pulses was 1600. This gave 160 s for the entire 3D data set and 1.6 s for a single projection image using a laser system with a 10-Hz pulse repetition rate. Piezoelectric array data acquisition required a minimum of two exposures of the sample at each orientation for the multiplexing. In our experiments, the number of averages was four, and the number of angular steps was 200, giving again a total of 1600 pulses. With a 20-Hz repetition rate, the time for gathering the data of a 3D image was therefore 80 s. The 3D image acquisition times that we achieved for both setups were too slow to avoid motion artifacts. However, both were limited by the pulse repetition rates of the lasers, where an acceleration by a factor of 10 is feasible. For both devices, data recording for a projection image without averaging can in principle be accomplished with a single laser exposure, allowing real time 2D tomography. For the piezoelectric array, the obvious modification would be to double the number of data acquisition channels. The camera setup currently needs two images to eliminate the background due to impurities and thermal gradients in the water bath, which could both be minimized with some modifications of the setup. Since the background image does not contain information about the acoustic field, it does not inhibit the capability of the device to create real-time projection images.

### 4.5. Manufacturing

Probably the most favorable property of the CCD camera setup is the extremely high number of densely distributed sensors (pixels). With a single exposure, it is able to create a data set with a size of about 1000 × 1000 values. A similar amount of information acquired with the piezoelectric array would require 1000 individual channels, each of them recording 1000 temporal samples. Here the camera is by far the more compact device.

The combination of optical excitation and acoustic detection is easier with the optical setup, where the actual sensor is acoustically and optically transparent. In the used configuration, where the sample was mounted on top of the water bath and illuminated from below, the initial pressure distribution in the object remained constant, even if the object was rotated. This was not the case for the piezoelectric array, where the sample had to be illuminated from both sides along the line direction. Since the object rotated relative to the illumination, the energy density distribution and the initial pressure changed at each rotational position.

Table 1 shows the differences and similarities of both tomographic setups. The camera setup wins in terms of the amount of data acquired per laser shot, while the piezoelectric array has an advantage due to its higher sensitivity. Concerning the operation of the device, piezoelectric detection enables the design of more user-friendly devices, which require minimal adjustments. Compared to other PAT setups with interferometric detection, the phase contrast approach is less susceptible to perturbations, making it a good choice for the implementation of optical sensing in a high-resolution diagnostic device.

## 5. Conclusions

Each of the presented imaging methods can be further improved, for instance by using more channels for the piezo array or by seeking ways to achieve higher sensitivity for the camera setup. The piezoelectric technology is more state of the art, and high-density arrays are readily available. Therefore, the transfer of this technology to dense line arrays is feasible. Consequently, it is more worthwhile to seek improvements of the camera setup to take advantage of the favorable properties of this sensing technique.

## Figures and Tables

**Table 1:** Differences and similarities of presented tomographic imaging setups. NEPLP: noise equivalent pressure-length product.

		Piezoelectric Array	Camera Setup
**Detection Bandwidth**		DC—12 MHz (non-uniform)	1.1–23 MHz (flat)
**Sensitivity (NEPLP)/Directivity**		0.4 kPa·mm/incident angle weighted response	5.1 kPa·mm/omnidirectional response
**Artifact Level**		High (streak artifacts, sparse sampling)	Low (dense spatial sampling)
**Image Resolution**		220 µm	80 µm
**Imaging Frame Rate**	**Projection**	capability for real-time, single shot imaging	
	**3D Imaging**	80 s ^1^	160 s ^1^
		about 2 s ^2^	
**User-friendliness**		high	moderate

^1^ Imaging frame rate of shown results. Data acquisition parameters are described in the operation section. ^2^ Possible achievable imaging frame rate using state-of-the-art laser systems with 100-Hz pulse repetition rate without performing averaging of raw signals.
